# Atypical Presentations of Neurosyphilis: The Great Imitator Revisited

**DOI:** 10.7759/cureus.88088

**Published:** 2025-07-16

**Authors:** Maryam Oulabasse, Yahya Naji, Khayer Sara, Loubna Chouaf, Sara Laadami, Nawal Adali

**Affiliations:** 1 Department of Neurology, University Hospital of Agadir, Agadir, MAR; 2 N.I.C.E. Research Team, R.E.G.N.E. Research Laboratory, Faculty of Medicine and Pharmacy, Ibn Zohr University, Agadir, MAR; 3 Department of Infectious Diseases, University Hospital of Agadir, Agadir, MAR

**Keywords:** atypical cases, great imitator, neurosyphilis, prevention, treponema pallidum

## Abstract

Background

Syphilis is a complex disease with a wide range of clinical manifestations. Neurosyphilis, a complication affecting the central nervous system, can occur at any stage of the disease, particularly in untreated cases. While classical forms are well documented, atypical presentations remain underrecognized, leading to diagnostic delays. This study explored atypical neurosyphilis cases at the Agadir University Hospital Center, Agadir, Morocco, emphasizing early recognition and management.

Methods

This retrospective study included patients with neurosyphilis for over two years. Ethical approval was obtained from all participants. All patients had a positive *Treponema pallidum* hemagglutination assay (TPHA) and cerebrospinal fluid (CSF) analysis. The diagnosis was based on either a reactive CSF Venereal Disease Research Laboratory (VDRL) test, a positive CSF TPHA test, or CSF lymphocytic pleocytosis associated with neurological symptoms. Data on demographics, clinical presentation, diagnostic findings, treatment, and outcomes were analyzed.

Results

Over a two-year period, 157 patients were diagnosed with syphilis, including 29 cases of neurosyphilis. The mean latency between primary syphilis and the onset of neurological symptoms was 20 years (range: 2-33 years). Meningovascular neurosyphilis (11 cases), chronic meningoencephalitis (6 cases), and tabes dorsalis (4 cases) were the most frequent classical forms. Seven (24.1%) patients exhibited atypical syndromes, including visual impairment, cerebellar ataxia, parkinsonism, sixth cranial nerve palsy, and epilepsy. Investigations confirmed a diagnosis of neurosyphilis. Treatment with intravenous ceftriaxone or penicillin led to clinical improvement.

Conclusion

Atypical neurosyphilis presentation complicates diagnosis, particularly in non-endemic areas. This study highlights the need to consider neurosyphilis in patients with unexplained neurological disorders, particularly in high-risk populations.

## Introduction

Syphilis, an infectious disease caused by the spirochete bacterium *Treponema pallidum*, is often referred to as the "great imitator" due to its wide-ranging and diverse clinical manifestations [1]. Neurosyphilis, a specific form of this disease, affects the central nervous system and can develop at any stage of syphilis, whether in the primary, secondary, or tertiary phases [1]. Historically, neurosyphilis has been reported to occur in approximately 4-10% of untreated syphilis cases, with an estimated annual incidence ranging from 0.16 to 2.1 cases per 100,000 individuals [2,3]. Invasion of the nervous system by *T. pallidum* often occurs early in the course of the infection, leading to a spectrum of possible outcomes. These range from spontaneous resolution to severe neurological damage caused by inflammatory infiltrates, obliterative endarteritis, and demyelination, which ultimately result in various clinical manifestations of neurosyphilis [4].

Traditionally, neurosyphilis is classified into five subtypes: early forms (asymptomatic, meningeal, and meningovascular neurosyphilis) and late forms (general paresis and tabes dorsalis, which typically manifest years or even decades after the initial infection) [5]. Diagnosis is based on a combination of patient history, clinical presentation, serological testing, and cerebrospinal fluid (CSF) analysis. Despite advancements in medical therapy, penicillin remains the cornerstone of treatment, with intravenous ceftriaxone (2 g/day) serving as an effective alternative for patients with penicillin allergies [6].

In recent years, there has been increasing recognition of atypical and oligosymptomatic presentations of neurosyphilis. These forms deviate from classical patterns and often manifest as isolated neurological syndromes including seizures, chronic headaches, cranial nerve involvement, isolated optic atrophy, and cerebellar ataxia. Such atypical presentations pose significant diagnostic challenges, particularly in regions with a high burden of infectious diseases where neurosyphilis may not be an immediate clinical consideration. Although these atypical forms are less frequently reported, emerging literature suggests that they are critical diagnostic considerations [7]. This study aims to emphasize the importance of early recognition and intervention and highlights the need for clinicians to consider neurosyphilis in patients presenting with unexplained neurological disorders, especially in high-risk populations. The objective is to improve diagnostic accuracy and management strategies for atypical forms of neurosyphilis.

## Materials and methods

This study involved a retrospective analysis of patients diagnosed with neurosyphilis over the past two years (January 2023 to December 2024) at the Agadir University Hospital Center, Agadir, Morocco. Ethical approval for this study was obtained from the Institutional Ethics Committee. All included patients had positive serum *Treponema pallidum* hemagglutination assay (TPHA) results and underwent CSF analysis. The diagnosis of neurosyphilis was established based on one of the following criteria: a positive CSF Venereal Disease Research Laboratory (VDRL) test, a positive CSF TPHA test, or the presence of CSF lymphocytic pleocytosis accompanied by relevant clinical symptoms.

Data collection included demographic characteristics, initial clinical presentation, and responses to treatment. Diagnostic and management procedures, including neuroimaging, CSF studies, and other relevant laboratory tests, were documented. The treatment protocols and patient outcomes following therapy were systematically recorded.

The diagnostic criteria for neurosyphilis adhered to the guidelines established by the Centers for Disease Control and Prevention (CDC) in both Europe and the United States [8,9]. According to these guidelines, neurosyphilis is diagnosed based on either (1) a reactive CSF VDRL test or (2) a non-reactive CSF VDRL test in conjunction with elevated CSF protein levels (>450 mg/L) or an increased CSF white blood cell count (>5 cells/μL), combined with neurological symptoms consistent with neurosyphilis, in the absence of an alternative explanation for the clinical presentation. Data were stored and analyzed using Microsoft Excel Version 2019 (Microsoft Corp., Redmond, WA). Descriptive statistics were employed to summarize demographic and clinical information.

## Results

**Figure 1:**
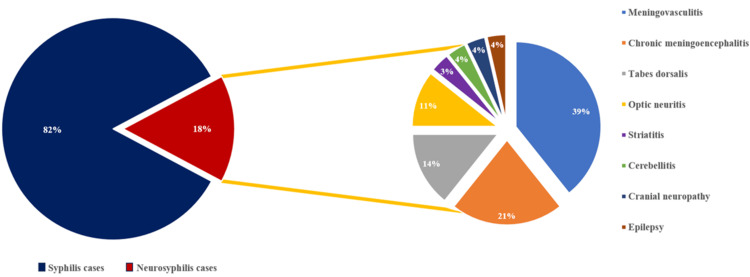
Neurosyphilis cases with the different presentations

Over a period of 2 years, 157 patients were diagnosed with syphilis, of whom 29 were diagnosed with neurosyphilis (Figure 1). One male patient reported engaging in sexual activity with other males. Fifty percent of the patients (15 patients) were from urban areas (Table 1). The mean latency period between the appearance of the chancre and the onset of neurological symptoms was 20 years (range: 2-33 years). The most common presentations of neurosyphilis were meningovasculitis (11 cases), chronic meningoencephalitis (6 cases), and tabes dorsalis (4 cases) (Figure 1).

**Table 1:** Clinical forms of neurosyphilis TPHA, *Treponema pallidum* hemagglutination assay; VDRL, Venereal Disease Research Laboratory

	Total neurosyphilis cases, n = 29	Atypical neurosyphilis cases, n = 7
Gender		
Male	28 (96%)	07 (25%)
Female	01 (4%)	00 (00%)
Mean age at diagnosis, year (range)	57.5 (33–82)	57 (45–69)
Sexual orientation		
Heterosexual	29 (100%)	07 (100%)
Homosexual	00 (51%)	00 (00%)
HIV status		
Positive	3 (10.5%)	1 (14%)
Negative	26 (89.5%)	6 (86%)
Serology at diagnosis (blood)		
TPHA mean titers (range)	10,280 (80–20,480)	
VDRL mean titers (range)	64 (negative to 128)	

Seven of the 29 patients with neurosyphilis exhibited atypical presentations. Detailed descriptions of each neurological syndrome, along with patient course, investigations, management, and outcomes, are summarized in Table 2.

**Table 2:** Clinical and paraclinical features of atypical neurosyphilis: summary of seven cases CSF, cerebrospinal fluid; VDRL, Venereal Disease Research Laboratory

Case no.	Age	Symptoms	Duration (months)	Neurological examination	MRI	CSF finding	VDRL in CSF	HIV Status	Time for improvement
1	47	Sudden decrease in binocular visual acuity	3	Reduced visual acuity	Optic atrophy	Normal	1_/8_	Negative	9 months
2	51	Photophobia and vision loss	6	Reduced visual acuity	Optic atrophy	Normal	½	Positive	6 months
3	51	Chronic headache and vision loss	9	Reduced visual acuity and nuchal rigidity	Optic neuritis	Lymphocytic meningitis	½	Positive	6 months
4	69	Progressive imbalance while walking	8	Gait ataxia and mild incoordination in both the upper and lower limbs.	Cerebellar atrophy	Normal	¼	Negative	6 months
5	61	Resting tremors in both upper limbs	12	Parkinsonian syndrome predominantly affecting the upper limbs	T2 signal abnormality in the bilateral basal ganglia	Normal	¼	Negative	9 months
6	50	Headaches and diplopia	1	Limited abduction of the left eye	Leptomeningeal and VI cranial nerve involvement	Lymphocytic meningitis	½	Negative	1 month
7	45	Afebrile epileptic seizures + of gastric discomfort and anorexia	3	Normal	Normal	Normal	½	Negative	3 months

Cases 1 to 3

Three patients developed optic neuropathy. A 47-year-old man with a history of high-risk sexual behavior and untreated syphilitic chancre 20 years ago, as well as a history of polysubstance abuse, presented with rapid bilateral visual acuity deterioration, rated at 20/200 in both eyes, over three months before hospitalization. Ophthalmological examination revealed bilateral optic atrophies. No significant neurological abnormalities were noted. Similarly, a 50-year-old man developed photophobia and bilateral vision loss, with a visual acuity of 20/200. A 51-year-old man presented with acute meningitis and bilateral visual acuity impairments (20/70). Neurological examination findings were unremarkable.

**Figure 2:**
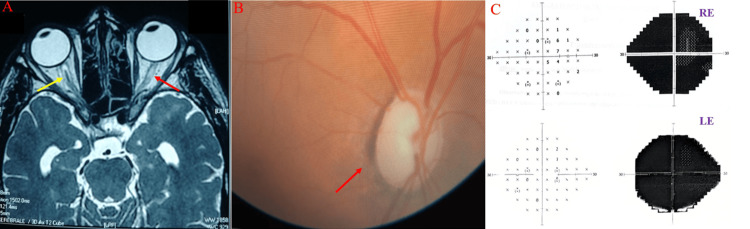
(A) MRI of the brain (T2 sagittal section) showing right optic atrophy with enlargement of the optic nerve sheath (yellow arrow) and hyperintense signals in the right optic nerve (red arrow). (B) Fundus examination showing cupping and pallor of the optic disc (red arrow). (C) Humphrey visual field test showing total vision loss in both eyes.

Three patients underwent complete investigations to rule out alternative diagnoses, including viral (herpes simplex virus and varicella zoster virus) and bacterial (tuberculosis and Lyme disease) infections, central retinal artery ischemia, intracranial tumors, and heavy metal poisoning. Serological tests confirmed syphilis, and CSF analysis revealed reactive VDRL (titers ranging from 1/8 to 1/2) and positive TPHA. Magnetic resonance imaging (MRI) revealed optic atrophy with hypersignals in the right optic nerve of the first patient (Figure 2).

The treatment consisted of intravenous penicillin G 24 million units per day for 14 days for the second patient and ceftriaxone 2 g daily for 14 days in addition to corticosteroid therapy. The first patient’s VDRL titer, initially 1/8, became negative after three treatment courses, whereas the VDRL titers of the other two patients, initially 1/2, became negative after a single course. Regarding visual acuity, only the third case showed partial recovery, with a result of 20/30 on follow-up.

Case 4

**Figure 3:**
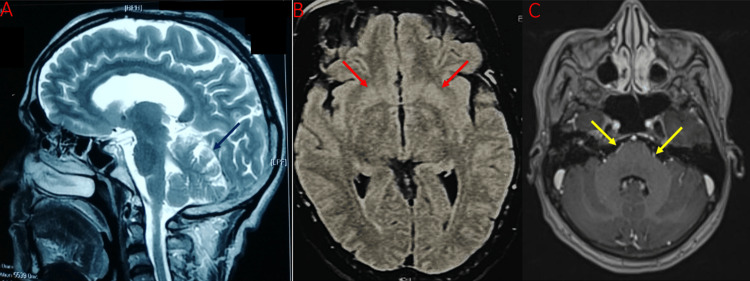
A. Brain MRI (T2 sagittal section) shows diffuse atrophy, predominantly in the olivo-ponto-cerebellar region, as observed in the fourth patient case (blue arrow). B. Brain MRI (T2 FLAIR coronal section) showing bilateral hyperintense signals in the striatum in the fifth patient case (red arrows). C. Brain MRI (CISS sequence) showing leptomeningeal and VI cranial nerve involvement in the sixth patient case (yellow arrows). CISS, constructive interference in steady state; FLAIR, fluid-attenuated inversion recovery

A 69-year-old male presented with progressive gait imbalance over the course of eight months, without any lateral preference. He denied any history of weakness or sensory deficits. Neurological examination revealed normal cognitive function, muscle tone, strength, and reflexes. However, significant gait ataxia and mild incoordination in both the upper and lower limbs were noted. Brain MRI demonstrated cortical and olivo-ponto-cerebellar atrophy (Figure 3A).

The differential diagnoses for this subacute progressive cerebellar syndrome included metabolic disorders (e.g., vitamin B12 deficiency, hypothyroidism), immune-mediated cerebellar ataxias (e.g., celiac disease, paraneoplastic syndromes, anti-GAD-associated ataxia), and chronic infections (e.g., fungal infections, central nervous system tuberculosis). The patient tested strongly positive for both serum and CSF VDRL and TPHA (CSF VDRL 1/4). CSF analysis revealed lymphocytic pleocytosis (20 cells/mm³) and an elevated protein level (62 mg/dL).

The patient was treated with intravenous ceftriaxone (2 g daily) for 14 days. Over the following six months, he exhibited significant improvement in gait and limb coordination, particularly following intensive rehabilitation sessions.

Case 5

A 61-year-old man with diabetes developed progressive resting tremors in both upper limbs over the course of one year. Neurological examination revealed generalized rigidity, bradykinesia, and exaggerated deep tendon reflexes, predominantly in the upper limbs. Saccadic eye movement testing showed no signs of slow or hypermetric saccades. Cognitive assessment indicated mild attention deficits, with no abnormalities noted in executive function or personality. Laboratory tests were unremarkable, including normal complete blood cell counts, electrolytes, renal and hepatic function, protein electrophoresis, and thyroid function. However, syphilis serology was positive. Cerebral MRI showed bilateral T2 signal abnormalities in the basal ganglia (Figure 3B). CSF analysis revealed normal protein levels without pleocytosis, a positive TPHA test, and a VDRL titer of 1/4.

The patient was treated with intravenous ceftriaxone (2 g daily) for 14 days and initiated on dopaminergic therapy. Following treatment, the patient demonstrated clinical improvement, with a subsequent negative VDRL titer, and remained on a daily dose of dopatherapy (500 mg per day).

Case 6

A 50-year-old man with type 2 diabetes presented with progressive headaches and rapidly worsening diplopia one month prior to hospitalization. The patient denied any vomiting, visual disturbances, focal neurological deficits, or seizures. He reported multiple unprotected sexual encounters with female partners. Neurological examination was unremarkable, except for the left abducens nerve palsy.

Cerebral MRI showed leptomeningeal and VI cranial nerve involvement (Figure 3C). The patient was referred to the neurology department, where a lumbar puncture revealed a normal opening pressure. Given positive syphilis serology in the blood, a CSF analysis was performed, demonstrating pleocytosis (87 cells/mm³, 92% lymphocytes), elevated protein levels (0.8 g/L), a positive TPHA, and a VDRL titer of 1/2. CSF Gram stain, Ziehl Nielsen stain, adenosine deaminase levels, tuberculosis (TB) polymerase chain reaction (PCR), and gene experts for TB were negative.

The patient was treated with intravenous penicillin G (24 million units/day for 14 days), without complications. The patient underwent gait training and physiotherapy. His headaches resolved, and his sixth cranial nerve palsy showed significant improvement within one month of treatment initiation.

Case 7

**Figure 4:**
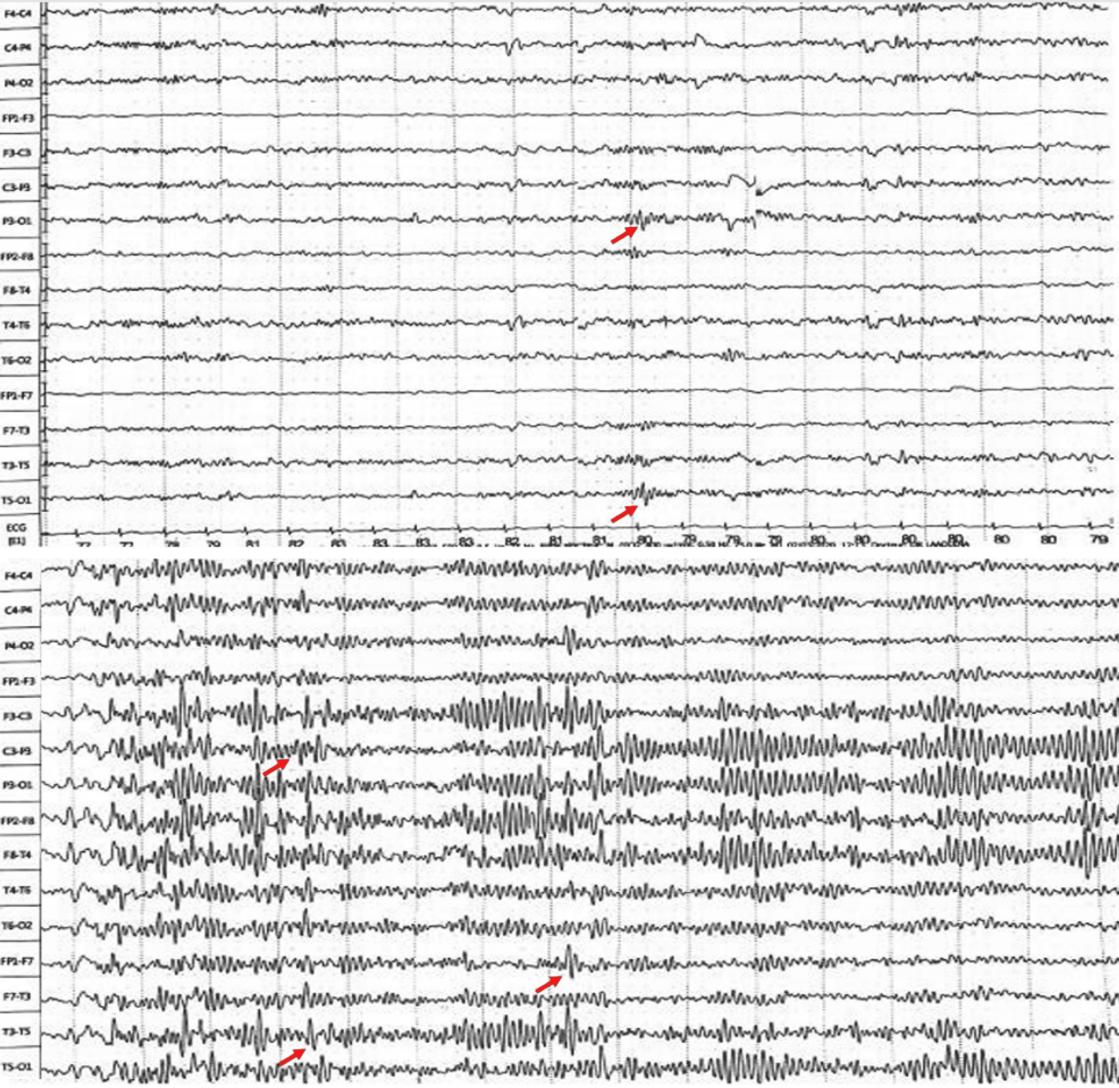
EEG showing paroxysmal generalized spike and spike-wave discharges, predominantly localized in the posterior regions (red arrows).

A 45-year-old man with no history of substance abuse, high-risk sexual behavior, or head trauma was admitted following sudden-onset afebrile epileptic seizures. Seizures were characterized by fixed gaze and oral automatisms, followed by transient aphasia. These symptoms were preceded by three months of gastric discomfort and anorexia. Neurological examination was unremarkable, and brain MRI showed no lesions. Initial metabolic and infectious workups (complete blood count and C-reactive protein) were within the normal limits. Electroencephalography (EEG) revealed continuous paroxysmal activity in the temporal cortex, leading to a diagnosis of mesiotemporal epilepsy (Figure 4).

Despite treatment with multiple antiseizure medications, the patient showed only minimal improvement. One year later, he reported that his wife had tested positive for syphilis, prompting his own testing. His syphilis serology returned positive results, and CSF analysis confirmed neurosyphilis, with a VDRL titer of 1/4. The patient received 2 g of ceftriaxone daily for 14 days and lamotrigine 100 mg per day. Within three months, he became seizure-free, demonstrating significant clinical recovery.

## Discussion

Neurosyphilis is a major medical concern, particularly in developing countries. Its association with HIV infection and the lack of adequate prevention strategies for sexually transmitted diseases have also contributed to an increasing number of cases in developed nations [10].

The Global Burden of Disease Study indicated that the global incidence of syphilis surged by 60% between 1990 and 2019, with approximately 50 million people infected worldwide in 2019 [11]. Before the advent of antibiotics, neurosyphilis was observed in approximately one-third of all syphilis cases [4]. Currently, it predominantly affects individuals with HIV, particularly those who are untreated, have low CD4+ counts, or exhibit detectable levels of HIV RNA [12]. The central nervous system is involved in approximately 14-20% of syphilis cases [13].

The demographic profile of our patients aligns with the existing literature: a predominance of males, rural backgrounds, low socioeconomic status, and Caucasian ethnicity. Notably, the likelihood of developing neurosyphilis is two to three times higher in white individuals than in black individuals despite black individuals exhibiting a five-fold higher incidence of other syphilis forms [14]. Furthermore, neurosyphilis is twice as prevalent in men as in women [4].

The high prevalence of rural patients in our study may be attributed to a lack of awareness of sexually transmitted diseases and limited access to health care facilities. These factors contribute to diagnostic delays, explaining the late-stage neurosyphilis observed in our study. In addition to the well-defined classical clinical forms, we also identified an increasing number of atypical presentations, likely due to inadequately administered penicillin treatments and delayed diagnoses.

Atypical forms of neurosyphilis are rarely reported in isolation and are often mixed with classical presentations in published case series. This study, which appears to be the second of its kind in Africa, highlights the importance of these atypical presentations [7].

In 1985, Burke and Schaberg analyzed 30 cases of neurosyphilis diagnosed in the United States between 1970 and 1981. Their findings revealed that 43% of cases presented with classic neurosyphilis symptoms, 14% were asymptomatic, and 43% exhibited nonspecific neurological disorders [15]. However, in our series, only 26.01% of the patients presented with atypical symptoms, and none were asymptomatic. Additionally, atypical manifestations of neurosyphilis have become increasingly prevalent, particularly among HIV-positive individuals and homosexual men. Between 1995 and 2005, 86% of neurosyphilis cases were atypical compared to 69% from 1965 to 1984. This shift highlights the evolving nature of neurosyphilis, with atypical forms now representing the dominant presentation. Diagnosis is challenging due to the variety of clinical symptoms and the absence of specific findings in imaging or CSF tests, emphasizing the need for early detection and treatment [16].

Optic atrophy

Optic atrophy, a known complication of neurosyphilis, was observed in 7.3% and 6.4% of cases in a series by Kissani et al. and Yahyaoui et al., respectively [17,18]. Similar results were reported in this study (7.69 %). Optic atrophy results from optic neuritis and can lead to progressive blindness over 5-10 years, significantly worsening the patient prognosis [18]. In syphilis, optic nerve involvement can be unilateral or bilateral and may present as perineuritis, anterior or retrobulbar optic neuritis, or papilledema [19]. While optic perineuritis is often asymptomatic, optic neuritis typically leads to rapid loss of vision [19]. However, in syphilitic cases, optic neuritis does not exhibit any distinctive features that differentiate it from other causes of optic nerve damage [20].

Parkinsonism and cerebellar lesion

Cerebellar involvement due to neurosyphilis is rarely documented in the literature [21]. In Kissani et al.’s series, cerebellar symptoms were observed in only 1.1% of cases [17]. As part of our study, a case report has been published describing mild cerebellar atrophy as well as brainstem atrophy and bilateral temporal lobe hyperintensities on T2/fluid-attenuated inversion recovery (FLAIR) [21].

Parkinsonian symptoms, characterized by tremors and rigidity due to basal ganglia involvement, were described as early as the 20th century by Guillain (1935) and Wilson (1940) [22]. Although previously reported, only a few cases of syphilis-associated parkinsonism exist, and the pathophysiological mechanism underlying striatal involvement remains poorly understood [23].

Seizure disorders

A meta-analysis published in 2021 highlighted that epileptic seizures are among the most frequently overlooked and misdiagnosed manifestations of syphilis, with prevalence rates ranging from 3.7% to 25.2% [24]. A South African study on status epilepticus in patients aged >13 years found that 8% had neurosyphilis [25]. Seizures may occur at various stages of the disease and can be attributed to small cerebral lesions resulting from microvascular accidents that do not produce overt neurological deficits. This aligns with the hypotheses regarding vascular epilepsy without clinically detectable neurological symptoms [26].

Cranial nerve palsy

Neurosyphilis cranial neuropathy occurs in approximately 6% of patients and affects the oculomotor, trigeminal, abducens, facial, and auditory nerves [27]. Isolated abducens nerve palsy is the most frequently reported condition [28]. Epidemiological studies have attributed isolated abducens palsy to neoplastic, traumatic, and microvascular causes, particularly in elderly patients. However, up to 30% of cases remain of undetermined etiology, possibly involving inflammatory, demyelinating, or infectious processes [29]. A meta-analysis published in 2022 identified 11 relevant studies that discussed the role of syphilis in abducens nerve involvement [29].

The treatment of adult-acquired syphilis, particularly neurosyphilis, prioritizes regimens that achieve treponemicidal antibiotic levels in CSF. Intravenous benzylpenicillin (18-24 million units/day for 10-14 days) remains the first-line treatment owing to its proven efficacy. Alternatives, such as ceftriaxone (1-2 g IV daily) or procaine penicillin combined with probenecid (when available), may be considered if IV therapy is not feasible. Doxycycline (100 mg twice daily or a single 200 mg dose orally) and azithromycin (2 g orally single dose) during 21-28 days serve as oral alternatives but require careful monitoring [30].

Primary care providers, who often serve as the first point of contact for patients with neurological symptoms, should maintain a high level of suspicion for neurosyphilis, particularly in high-risk populations. Neurologists, on the other hand, are likely to face the challenge of diagnosing and managing these cases once symptoms have progressed. A multidisciplinary approach, involving infectious disease specialists, neurologists, and primary care physicians, is essential for effective diagnosis and management.

Despite the valuable insights provided by this study, several limitations must be acknowledged. The sample size was relatively small, as it was based on a single-center, retrospective analysis, which may limit the generalizability of the findings. The retrospective nature of the study also introduces the possibility of selection bias and incomplete data. Furthermore, the study’s reliance on historical records may lead to variability in diagnostic practices and treatment protocols over time. These limitations suggest that future multi-center, prospective studies with larger sample sizes are needed to confirm our findings and improve the understanding of atypical neurosyphilis presentations.

## Conclusions

In addition to the rising global incidence and diagnostic delays owing to limited access to care, the atypical clinical presentation of neurosyphilis further complicates patient management. Despite an oligosymptomatic clinical picture, syphilis serology testing should be conducted in both blood and CSF when risk factors are present. This “atypical” group likely represents an overlap of conventional neurosyphilis classifications. The diagnosis and treatment remain the same as those for other neurosyphilis subtypes, emphasizing the importance of early detection and multidisciplinary management.
